# An Epidemiologic Review of Enteropathogens in Gaborone, Botswana: Shifting Patterns of Resistance in an HIV Endemic Region

**DOI:** 10.1371/journal.pone.0010924

**Published:** 2010-06-02

**Authors:** Jack S. Rowe, Samir S. Shah, Stephen Motlhagodi, Margaret Bafana, Ephraim Tawanana, Hong T. Truong, Sarah M. Wood, Nicola M. Zetola, Andrew P. Steenhoff

**Affiliations:** 1 Botswana-UPenn Partnership, Gaborone, Botswana; 2 University of Pennsylvania School of Medicine, Philadelphia, Pennsylvania, United States of America; 3 Botswana National Health Laboratory, Gaborone, Botswana; 4 Princess Marina Hospital, Gaborone, Botswana; 5 The Children's Hospital of Philadelphia, Philadelphia, Pennsylvania, United States of America; University of Cape Town, South Africa

## Abstract

**Background:**

The epidemiology of diarrheal disease in Botswana, an HIV endemic region, is largely unknown. Our primary objective was to characterize the prevalent bacterial and parasitic enteropathogens in Gaborone, Botswana. Secondary objectives included determining corresponding antimicrobial resistance patterns and the value of stool white and red blood cells for predicting bacterial and parasitic enteropathogens.

**Methodology/Principal Findings:**

A retrospective cross-sectional study examined laboratory records of stool specimens analyzed by the Botswana National Health Laboratory in Gaborone, Botswana from February 2003 through July 2008. In 4485 specimens the median subject age was 23 [interquartile range 1.6–34] years. Overall, 14.4% (644 of 4485) of samples yielded a pathogen. Bacteria alone were isolated in 8.2% (367 of 4485), parasites alone in 5.6% (253 of 4485) and both in 0.5% (24 of 4485) of samples. The most common bacterial pathogens were *Shigella* spp. and *Salmonella* spp., isolated from 4.0% (180 of 4485) and 3.9% (175 of 4485) of specimens, respectively. *Escherichia coli* (22 of 4485) and *Campylobacter* spp. (22 of 4485) each accounted for 0.5% of pathogens. Comparing antimicrobial resistance among *Shigella* spp. and *Salmonella* spp. between two periods, February 2003 to February 2004 and July 2006 to July 2008, revealed an increase in ampicillin resistance among *Shigella* spp. from 43% to 83% (p<0.001). Among *Salmonella* spp., resistance to chloramphenicol decreased from 56% to 6% (p<0.001). The absence of stool white and red blood cells correlated with a high specificity and negative predictive value.

**Conclusions/Significance:**

Most gastroenteritis stools were culture and microscopy negative suggesting that viral pathogens were the majority etiologic agents in this Botswana cohort. *Shigella* spp. and *Salmonella* spp. were the most common bacteria; *Isospora* spp. and *Cryptosporidium* spp. were the most common parasites. Resistance to commonly used antimicrobials is high and should be closely monitored.

## Introduction

Diarrheal disease is a serious cause of mortality and morbidity in Sub-Saharan Africa, accounting for an estimated 16% of deaths in Africa among children <5 years of age[1]. The burden of diarrheal disease is amplified by Africa's human immunodeficiency virus (HIV) epidemic, as diarrheal disease is a major cause of mortality and morbidity among HIV-infected patients and can intensify HIV-related wasting and malnutrition [2]. HIV-infected subjects have a predilection for chronic diarrhea, which is most pronounced in those with lowest CD4+ cell counts [3], [4].

A broad range of etiologic agents are responsible for acute and chronic diarrheal disease, and the prevalence of such agents varies greatly by geographic region, season, patient age, immune status, and socioeconomic conditions. The dynamic variability of etiologic agents has been shown in studies throughout southern Africa [5], [6], [7], [8], [9], [10], [11], [12]. Several Sub-Saharan African studies have also indicated a high prevalence of resistance to commonly used antimicrobials, such as ampicillin and trimethoprim-sulfamethoxazole [5], [8], [9], [12]. However, resistance patterns are often regionally-specific, and there is little data describing how these patterns have changed over time. To date, there is limited data regarding the epidemiology of diarrheal disease in Gaborone, Botswana, an HIV endemic region.

The primary objective of this study was to determine the prevalence of bacterial and parasitic enteropathogens in Gaborone, Botswana in stool samples from both inpatient and outpatient adult and pediatric populations. Secondary objectives were to describe antimicrobial susceptibilities of the most frequently occurring bacterial pathogens, *Salmonella* spp. and *Shigella* spp., and to determine the sensitivity, specificity, positive predictive value and negative predictive value of stool white blood cells (WBC) and red blood cells (RBC) for bacterial and parasitic enteropathogens.

## Methods

### Ethics Statement

This study was reviewed and approved by the institutional review boards of the Botswana Ministry of Health (Gaborone, Botswana), Princess Marina Hospital (Gaborone, Botswana), and The Children's Hospital of Philadelphia (Philadelphia PA, USA). A waiver of informed consent was granted by all review boards given that the study represented a de-identified, retrospective study of routine clinical practice with no more than minimal risk to subjects.

### Study Design, Setting, and Participants

A retrospective, cross-sectional study of stool specimen records collected between February 1, 2003 and July 31, 2008 was performed at the Botswana National Health Laboratory (BNHL) in Gaborone, Botswana. The BNHL, which serves a population of 500,000, is the reference microbiology laboratory for the public health facilities in and those surrounding Gaborone, Botswana. Stool samples received from Princess Marina Hospital (PMH), the largest tertiary care referral center in Botswana, as well as from clinics and smaller hospitals within a 30 km radius of Gaborone were eligible for inclusion. Specimens were excluded if they came from a patient without gastroenteritis. Botswana has the second highest prevalence of HIV in the world. In 2007, an estimated 23.9% of Batswana aged 15–49 years were HIV positive [13].

### Microbiology Methods

Stool samples were subjected to microscopy, culture, and antimicrobial susceptibility testing. Routine laboratory practice for stool samples at the BNHL during the study period followed a standard operating procedure including a 24 hour turn-around-time for stool microscopy and processing of all stool samples within 24 hours of collection. All samples were assessed by a qualified laboratory technologist who was supervised by a laboratory scientist. Weekend coverage included a technologist on duty until 4 pm each day. Antimicrobial susceptibility patterns of isolates were determined by disk-diffusion method according to Clinical Laboratory Standards Institute (CLSI) guidelines[14]. Prior to October 2003, BNHL routinely tested stool *Salmonella* spp. and *Shigella* spp. for susceptibility to ampicillin, trimethoprim-sulfamethoxazole, gentamicin, trimethoprim, tetracycline, cefotaxime, and ampicillin-sulbactam. From October 2003, BNHL adopted the World Health Organization (WHO) recommended panel of susceptibility testing to ampicillin, trimethoprim-sulfamethoxazole, ciprofloxacin, chloramphenicol, and nalidixic acid [15]. Apart from this change in susceptibility testing, other laboratory practices remained unchanged during the course of the study.

### Data Collection and Statistical Analysis

Electronic and paper-based records of the BNHL were reviewed to identify stool samples meeting study inclusion criteria. Demographic data abstracted included age, sex and, for inpatient samples only, ward location. Results of antimicrobial susceptibility testing were recorded when available. In addition, exposure to antibiotics in the 2 weeks before the stool sample was submitted was recorded as antibiotic exposure in this period may have influenced the antimicrobial susceptibilities of bacterial pathogens. Consistent and complete stool records were available for two selected study periods: 1^st^ February 2003 through 27^th^ February 2004 and 1^st^ July 2006 through 31^st^ July 2008. For the period 28^th^ Feb 2004 to 30^th^ June 2006, records were available for 11 (39.3%) of 28 months. This period contributed 308 (6.9%) of 4485 study specimens. While the overall analysis included all specimens analyzed from February 2003 through July 2008, the two selected study periods were compared to determine whether changes in antimicrobial susceptibility patterns occurred over time. Overall proportions of different pathogens found in stool were calculated and stratified as a measure of disease burden.

Data were analyzed using STATA version 9.2 (Stata Corp., College Station, TX). Categorical variables were compared using Fisher exact tests. Sensitivity, specificity, positive predictive value and negative predictive value were calculated to determine the accuracy of stool white blood cells and red blood cells in predicting bacterial and parasitic infections.

## Results

### Characteristics of the Study Population

During the study period, 90.4% (4485 of 4960) of stool samples met inclusion criteria. Samples were excluded because no result was recorded (205 of 4960) or the sample came from a patient without gastroenteritis (270 of 4960). Outpatient services (Gaborone city clinics, PMH outpatients and other clinics within 30 km of Gaborone) accounted for 70.5% (3162 of 4485) of specimens included in this study. Samples from inpatients at PMH accounted for 26.7% (1197 of 4485) of specimens. The location was unknown for 2.8% (126 of 4485) of specimens. The demographic characteristics of patients from which samples were received are described in **Table 1**. The median subject age was 23 years [interquartile range: 1.6–34 years].

**Table 1:** Demographic Characteristics of Included Stool Specimens*.

	Gender**		Age Group							
	Female (n = 2255)	Male (n = 1870)	<1 mo (n = 95)	≥1 mo – <12 mo (n = 629)	≥1 yr – <5 yr (n = 564)	≥5 yr – <15 (n = 313)	≥15 yr – <50 yr (n = 2166)	≥50 yr – <65 yr (n = 185)	≥65 yr (n = 77)	Unknown age (n = 456)
**Samples Positive for Bacteria (n = 367)**	171 (8)	171 (9)	4 (4)	61 (10)	64 (11)	30 (10)	162 (7)	12 (6)	5 (6)	29 (6)
**Samples Positive for Parasites (n = 253)**	123 (5)	111 (6)	1 (1)	23 (3)	43 (8)	20 (6)	122(6)	12 (6)	2 (3)	30 (7)
**Samples Positive for both Bacteria and Parasites (n = 24)**	11 (<1)	12 (<1)	0 (0)	5 (1)	9 (2)	1(<1)	8 (<1)	1 (<1)	0 (0)	0 (0)
**Samples with no pathogen isolated (n = 3841)**	1950 (86)	1576 (84)	90 (95)	540 (86)	448 (79)	262 (84)	1874 (87)	160 (86)	70 (91)	397 (87)

*values listed as number (percent of specimens in gender or age group).

**data on sex missing for 25 samples positive for bacteria, 19 samples positive for parasites, 1 sample positive for both bacteria and parasites, and 315 samples with no pathogen identified.

### Epidemiologic review of pathogens

Overall, 14.4% (644 of 4485) of samples yielded a pathogen. Bacteria alone were isolated in 8.2% (367 of 4485), parasites alone in 5.6% (253 of 4485) and both parasites and bacteria in 0.5% (24 of 4485). Of the 367 samples that isolated bacteria alone, 8 samples isolated two types of pathologic bacteria. Because of this, the total number of bacterial isolates is 399 (367+24+8). The most common bacterial pathogens were *Shigella* spp. and *Salmonella* spp., isolated from 4.0% (180 of 4485) and 3.9% (175 of 4485) of all specimens, respectively. *Escherichia coli* (22 of 4485) and *Campylobacter* spp. (22 of 4485) each accounted for 0.5% of all specimens. Of the *Shigella* spp., *S. flexneri* was the most common, accounting for 63.3% (114 of 180) of all *Shigella* isolates, followed by *S. sonnei* (15.6%), *S. dysenteriae* (11.1%), and *S. boydii* (7.2%). Data for specific serotypes of *Salmonella* spp. were not available, other than for two cases of *S. typhi*.

The most common parasites were *Isospora* spp. and *Cryptosporidium* spp., found in 2.5% (113 of 4485) and 2.2% (99 of 4485) of all specimens, respectively. Other common parasites were *Giardia lamblia* (0.8%) and *Taenia* spp. (0.6%).

Individual pathogens were stratified by patient age and selected study period as illustrated in **Table 2** and **Table 3** respectively. The association of WBC or RBC with the presence of bacterial isolates or parasites is depicted in **Table 4**.

**Table 2:** Proportion of pathogens by age group*.

Pathogen	Age Group [n (%)]**							
	<1 mo (n = 95)	≥1 mo – <12 mo (n = 629)	≥1 yr – <5 yr (n = 564)	≥5 yr – <15 yr (n = 313)	≥15 yr – <50 yr (n = 2166)	≥50 yr – <65 yr (n = 185)	≥65 yr (n = 77)	Unknown age (n = 456)
BACTERIA								
*Salmonella* spp.	1 (1)	34 (5)	29 (5)	13 (4)	80 (4)	5 (3)	2 (3)	11 (2)
*Escherichia coli*	2 (2)	11 (2)	9 (2)	0 (0)	0 (0)	0 (0)	0 (0)	0 (0)
*Campylobacter* spp.	2 (2)	8 (2)	8 (1)	2 (<1)	2 (<1)	0 (0)	0 (0)	0 (0)
*Shigella* spp.	0 (0)	14 (2)	29 (5)	16 (5)	92 (4)	8 (4)	3 (4)	18 (4)
**Total bacterial isolates:**	5 (5)	67 (11)	75 (13)	31 (10)	174 (8)	13 (7)	5 (6)	29 (6)
PARASITES								
*Cryptosporidium* spp.	0 (0)	25 (4)	42 (7)	3 (1)	17 (<1)	7 (4)	1 (1)	4 (1)
*Isospora* spp.	1 (1)	1 (<1)	3 (1)	7 (2)	78 (4)	4 (2)	0 (0)	19 (4)
*Giardia lamblia*	0 (0)	2 (<1)	6 (1)	7 (2)	13 (<1)	1 (<1)	1 (1)	4 (1)
Other Parasites	0 (0)	0 (0)	1 (<1)	4 (1)	24 (1)	1 (<1)	0 (0)	3 (<1)
**Total parasitic isolates**	1 (1)	28 (4)	52 (9)	21 (7)	132 (6)	13 (7)	2 (3)	30 (7)
NO PATHOGEN	90 (95)	540 (86)	448 (79)	262 (83)	1874 (87)	160 (86)	70 (91)	397 (87)

Abbreviations: mo, month(s); yr, year(s); spp, species.

*values listed as number (percent of specimens in age group).

**not all columns sum to 100% due to co-infection with multiple pathogens among some specimens.

**Table 3:** Comparison of pathogen proportions between selected study periods.

Pathogen	Feb 2003-Feb 2004 Isolates# (n = 1332)**	Jul 2006-Jul 2008 Isolates# (n = 2845)**	p-value*
**BACTERIA**			
*Salmonella* spp.	48 (4)	110 (4)	0.728
*Escherichia coli*	5 (<1)	17 (<1)	0.492
*Campylobacter* spp.	10 (<1)	12 (<1)	0.175
*Shigella* spp.	74 (6)	97 (3)	**0.001**
Total bacterial isolates:	137 (10)	236 (8)	**0.041**
**PARASITES**			
*Cryptosporidium* spp.	10 (<1)	76 (3)	**<.001**
*Isospora* spp.	42 (3)	69 (2)	0.180
*Giardia lamblia*	21 (2)	10 (<1)	**<.001**
Other Parasites	5 (<1)	25 (1)	0.079
Total parasitic isolates:	78 (6)	180 (6)	0.582
NO PATHOGEN	1124 (84)	2450 (86)	0.143

# Number (and %) of specimens in selected study period.

*p-values calculated using two-tailed Fisher Exact Test.

**not all columns sum to 100% due to co-infection with multiple pathogens among some specimens.

**Table 4:** Association of WBC and RBC with the presence of bacteria or parasites.

	Bacteria Present	Parasites Present
**Presence of White Blood Cells**		
Sensitivity	54.0%	30.6%
Specificity	74.2%	69.9%
Positive Predictive Value	27.4%	10.8%
Negative Predictive Value	90.0%	89.4%
**Presence of Red Blood Cells**		
Sensitivity	21.4%	3.7%
Specificity	92.2%	89.4%
Positive Predictive Value	33.2%	4.0%
Negative Predictive Value	86.7%	88.6%
**Presence of Both White and Red Blood Cells**		
Sensitivity	21.5%	3.7%
Specificity	92.9%	90.1%
Positive Predictive Value	35.2%	4.3%
Negative Predictive Value	86.9%	88.7%

Abbreviations: WBC, white blood cells; RBC, red blood cells; WBC or RBC were counted as present if laboratory records indicated scanty, few, moderate, or many cells upon microscopic evaluation.

### Antimicrobial Susceptibility

Susceptibility data were available for 87.7% (350 of 399) of positive bacterial isolates. Because data were most consistently available for susceptibility to ampicillin, trimethoprim-sulfamethoxazole, chloramphenicol, and nalidixic acid within the two selected study periods, resistance patterns to these antimicrobials for all bacterial isolates were compared (**Table 5**). There was a significant increase in resistance to ampicillin among *Shigella* spp. isolates and a significant decrease in resistance to chloramphenicol among *Salmonella* spp. isolates over time. No *Salmonella* spp. or *Shigella* spp. isolates were resistant to ciprofloxacin, while 0% (0 of 10) and 22% (2 of 9) *Campylobacter* spp. were resistant to ciprofloxacin in the earlier and later selected study periods, respectively. When all bacterial isolates were pooled together, significant findings were an increase in ampicillin resistance (p<0.001), and decreased resistance to trimethoprim-sulfamethoxazole (p = 0.028) and chloramphenicol (p = 0.001).

**Table 5:** Comparison of antibiotic resistance among *Salmonella* spp., *Shigella* spp., and all bacterial isolates.

Antibiotic	Feb 2003-Feb 2004 Resistant Isolates#	Jul 2006-Jul 2008 Resistant Isolates#	p-value*
***Salmonella*** ** spp.**			
Ampicillin	19/46 (41)	43/87 (49)	0.465
Trimethoprim-Sulfamethoxazole	16/46 (35)	19/86 (22)	0.148
Chloramphenicol	14/25 (56)	5/82 (6)	**<.001**
Nalidixic Acid	4/25 (16)	22/87 (25)	0.426
***Shigella*** ** spp.**			
Ampicillin	32/74 (43)	66/80 (83)	**<.001**
Trimethoprim-Sulfamethoxazole	60/74 (81)	62/80 (78)	0.692
Chloramphenicol	6/16 (38)	21/79 (27)	0.378
Nalidixic Acid	0/16 (0)	8/80 (10)	0.345
**All Bacterial Isolates (** ***Salmonella, Shigella*** **, ** ***Campylobacter*** **, ** ***E. coli*** **)** †			
Ampicillin	56/127 (44)	125/189 (66)	**<.001**
Trimethoprim-Sulfamethoxazole	82/127 (65)	97/187 (52)	**0.028**
Chloramphenicol	21/51 (41)	31/182 (17)	**0.001**
Nalidixic Acid	6/43 (14)	37/189 (20)	0.515

# Number (and %) of organisms resistant.

*p-values calculated using two-tailed Fisher Exact Test.

†This section includes all bacterial isolates (*Salmonella spp.*and *Shigella spp.*as well as the few isolates of *Campylobacter spp.* and *E.coli*). This summary section may be a useful guide to empiric therapy of dysentery in Southern Botswana.

Of 644 specimens that yielded a pathogenic organism, data regarding previous antimicrobial exposure within two weeks of specimen collection were available for 22% (139 of 644). Of these, 24% (33 of 139) were exposed to antimicrobials within 2 weeks as follows: cefotaxime 33% (11 of 33), trimethoprim-sulfamethoxazole 30% (10 of 33), and metronidazole 24% (8 of 33).

## Discussion

Our study reports a high proportion of stool specimens with no identifiable pathogenic bacteria or parasites. When pathogens were identified, *Shigella* spp. and *Salmonella* spp. were the most common bacteria, while *Isospora* spp. and *Cryptosporidium* spp. were the most common parasites. We also identified important trends in antimicrobial susceptibility among common agents responsible for gastroenteritis in southern Africa. The proportion of resistance to ampicillin and trimethoprim-sulfamethoxazole among common pathogens was high, supporting the utility of nalidixic acid as empiric therapy for suspected bacterial dysenteric gastroenteritis. Although significant changes in resistance to nalidixic acid were absent, susceptibility to this agent should be closely monitored.

Compared with previous studies of diarrheal disease in the region, our data showed a markedly lower overall proportion of bacterial and parasitic isolates. These differences could be partially attributed to the fact that few other regional studies comprehensively evaluated a similar breadth of pathogens, and most were restricted to children. In our study, if we restricted the proportion analysis of bacterial pathogens to children younger than 5 years of age, the rate increases to 11.1% (143 of 1288), although this is still lower than other regional studies. Studies from Zimbabwe, Mozambique, South Africa, and Kenya have reported bacterial pathogens in 22% to 32% of specimens from patients with diarrheal disease [5], [8], [16], [17]. The same studies from Mozambique and South Africa also reported that 11 to 14% of specimens contained a parasite [8], [16]. In our study, parasite proportion analysis restricted to children under 5 years of age showed an increased, although still discordant, number of 6.3% (67 of 1288) of specimens contained a parasite.

The proportion of specimens positive for *Shigella* spp. was also substantially lower than most other estimates in the region, which ranged from 10–16% [5], [12], [17], [18], [19]. However, a study in southern Mozambique of children <5 years with diarrheal disease indicated a lower proportion of *Shigella* spp. of 0.2% [8]. The antimicrobial susceptibilities we describe for *Shigella* isolates are similar to those previously reported from the region with ranges of resistance reported at 77–97% for ampicillin, 90–97% for trimethoprim-sulfamethoxazole, 27–88% for chloramphenicol, 0–3% for ciprofloxacin, and 0–2% for nalidixic acid [5], [9], [12].

In the present study, the proportion of *Salmonella* spp. was similar to other estimates from the region, which ranged from 1.4–5.8% [5], [8], [17], [18], [19]. In addition, the percent of resistance among *Salmonella* isolates was similar to regional studies with ranges of 13–62% for ampicillin, 4–88% for trimethoprim-sulfamethoxazole, 3–36% for chloramphenicol, 0–1% for ciprofloxacin and 3–33% for nalidixic acid) [5], [9], [12], [17].

We also reported a lower overall proportion of *Campylobacter* spp., *E. coli*, *Isospora* spp., *Cryptosporidium* spp., and *G. lamblia*, than have been seen in other studies in the region. For these pathogens, other Sub-Saharan Africa studies have indicated ranges of: *Campylobacter* spp. (1–9%), *E. coli* (2–23%), *Isospora* spp.(12%), *Cryptosporidium* spp. (0.5–16%), and *G. lamblia* (1–7%) [5], [6], [8], [16], [17], [18], [19], [20], [21], [22].

The calculated sensitivity, specificity, positive predictive value, and negative predictive value of WBC and/or RBC in determining the presence of bacteria were within reported ranges of previous studies examining acute infectious diarrhea [23]. In this setting, the absence of WBC and/or RBC was generally correlated with a high specificity and negative predictive value for the absence of bacteria or parasites.

The only similar study concerning diarrheal disease in Gaborone included 221 children with diarrhea enrolled prospectively from July through November, 1998 at a single clinic serving a relatively socioeconomically poor area [24]. The 21% prevalence of *Shigella* spp. in that study was higher than the present study (4.0% of all samples); 89% of isolates were resistant to ampicillin and 39% were resistant to trimethoprim-sulfamethoxazole. The prevalence of *Salmonella* spp., was similar to that found in our study and, in contrast to our results, all *Salmonella* spp. were sensitive to ampicillin and trimethoprim-sulfamethoxazole. While prospective, the *Urio et al* study was limited to a five month period, did not examine antimicrobial resistance in enteropathogens other than *Salmonella* or *Shigella* and reflects a single clinic pediatric experience in a low socioeconomic setting [24].

There are several possible explanations for the discrepancy in enteropathogen prevalence rates between this study and others previously discussed. The majority of specimens in this study are from outpatient clinics, while many previous studies were restricted to inpatient admissions. Inpatient samples may select for more severe cases of diarrhea and thus bias those studies toward a higher prevalence of bacterial pathogens [8], [16]. Because of the retrospective design, we could not control for the amount of time between specimen collection and analysis; this may have biased our results towards a larger proportion of samples being negative for bacteria and/or parasites, as some bacteria (e.g. *Shigella, Campylobacter*) and many parasites are sensitive to desiccation when left in the specimen container for an extended period of time. This bias would cause us to underestimate the prevalence of some pathogens, but would not impact the interpretation of susceptibility data. Because Botswana has one of the highest HIV prevalence rates in Africa [13], there may also be a larger proportion of negative specimens due to a relatively higher prevalence of HIV enteropathy. Some regional variation in pathogen prevalence is also expected, as climate, seasonality, and socioeconomic conditions are influential. In addition, the water supply and sanitation in the study area are relatively good. This makes contamination of drinking water by bacteria or parasites less likely thereby decreasing their proportional contribution as a cause of gastroenteritis.

This study had several limitations. Our retrospective and descriptive design makes our results susceptible to all limitations and potential biases of studies of similar design. We were unable to account for multiple specimens from the same patient, which precluded incidence rate calculation. Data on recent antimicrobial exposure were not routinely available, although those samples for which data were available indicated that the percentage of specimens previously exposed to antimicrobials was relatively low. Due to the retrospective study design, standard laboratory techniques and data recording practices shifted over the course of the study period. Because of changes in laboratory practices, isolates of *Salmonella* and *Shigella* were submitted to different susceptibility testing panels before and after October 2003. It has also been laboratory practice not to routinely differentiate *Cyclospora* from *Cryptosporidium*; thus, the prevalence of *Cryptosporidium* may be lower than reported in this study. However, there have been few reported cases of *Cyclospora* in Sub-Saharan Africa, and we believe this contribution to be negligible [20], [25]. We were unable to obtain the HIV status of patients from whom stool specimens were submitted. HIV itself may predispose our patient population to specific pathogens in this HIV endemic region and thus limit the generalizability of our data. Our study does, however, encompass a longer time period and larger sample size than other reports from the region [5]. Additionally, we describe antimicrobial resistance patterns over time and, by including both inpatient and outpatient specimens from a wide variety of centers, we limited the referral bias likely present in other studies that examined only inpatients with diarrhea. Both of these aspects are novel for data from the southern African region.

In summary, this study demonstrates a high prevalence of samples negative for bacteria and parasites, likely indicating a high prevalence of viral illness although further prospective studies are needed to confirm such findings. *Shigella* spp. and *Salmonella* spp. were the most common bacteria; *Isospora* spp. and *Cryptosporidium* spp. were the most common parasites. Resistance to commonly used antimicrobials among enteropathogens in Gaborone, Botswana and the surrounding area is high. Nalidixic acid may provide the best alternative for empiric therapy in a patient with dysentery, although such use should be closely monitored as resistance to nalidixic acid is also increasing.
